# Harm reduction principles for healthcare settings

**DOI:** 10.1186/s12954-017-0196-4

**Published:** 2017-10-24

**Authors:** Mary Hawk, Robert W. S. Coulter, James E. Egan, Stuart Fisk, M. Reuel Friedman, Monique Tula, Suzanne Kinsky

**Affiliations:** 1 Department of Behavioral and Community Health Sciences, Graduate School of Public Health, University of Pittsburgh, 4136 Parran Hall, Pittsburgh, USA; 2 School of Medicine, University of Pittsburgh, 3414 Fifth Avenue, Pittsburgh, PA 15213 USA; 3 Behavioral and Community Health Sciences, Graduate School of Public Health, University of Pittsburgh, 4138 Parran Hall, Pittsburgh, USA; 4 Center for Inclusion Health, West Penn Allegheny Health Network, Pittsburgh, PA USA; 5 Infectious Diseases and Microbiology, Graduate School of Public Health, University of Pittsburgh, 400 Keystone Building, Pittsburgh, USA; 6 Harm Reduction Coalition, 1440 Broadway, Oakland, CA 94602 USA; 7 UPMC Center for High Value Healthcare, U.S. Steel Tower, 600 Grant St., 40th Floor, Pittsburgh, PA 15219 USA

## Abstract

**Background:**

Harm reduction refers to interventions aimed at reducing the negative effects of health behaviors without necessarily extinguishing the problematic health behaviors completely. The vast majority of the harm reduction literature focuses on the harms of drug use and on specific harm reduction strategies, such as syringe exchange, rather than on the harm reduction philosophy as a whole. Given that a harm reduction approach can address other risk behaviors that often occur alongside drug use and that harm reduction principles have been applied to harms such as sex work, eating disorders, and tobacco use, a natural evolution of the harm reduction philosophy is to extend it to other health risk behaviors and to a broader healthcare audience.

**Methods:**

Building on the extant literature, we used data from in-depth qualitative interviews with 23 patients and 17 staff members from an HIV clinic in the USA to describe harm reduction principles for use in healthcare settings.

**Results:**

We defined six principles of harm reduction and generalized them for use in healthcare settings with patients beyond those who use illicit substances. The principles include humanism, pragmatism, individualism, autonomy, incrementalism, and accountability without termination. For each of these principles, we present a definition, a description of how healthcare providers can deliver interventions informed by the principle, and examples of how each principle may be applied in the healthcare setting.

**Conclusion:**

This paper is one of the firsts to provide a comprehensive set of principles for universal harm reduction as a conceptual approach for healthcare provision. Applying harm reduction principles in healthcare settings may improve clinical care outcomes given that the quality of the provider-patient relationship is known to impact health outcomes and treatment adherence. Harm reduction can be a universal precaution applied to all individuals regardless of their disclosure of negative health behaviors, given that health behaviors are not binary or linear but operate along a continuum based on a variety of individual and social determinants.

## Background

Harm reduction refers to interventions aimed at reducing the negative effects of health behaviors without necessarily extinguishing the problematic health behaviors completely or permanently. Though the harm reduction model as we know it rose in prominence in the1970s and 1980s in response to infectious diseases such as hepatitis B and HIV [1], its roots extend at least as far back as the early 1900s with narcotic maintenance clinics [2, 3]. In the context of substance use, harm reduction disentangles the notion that drug use equals harm and instead identifies the negative consequences of drug use as the target for intervention rather than drug use itself [4]. Harm reduction strategies include syringe exchange programs, safer injection facilities, overdose prevention programs and policies, and opioid substitution treatment. Harm reduction as an approach stands in opposition to the traditional medical model of addiction which labels any illicit substance use as abuse, as well as to the moral model, which labels drug use as wrong and therefore illegal [5]. While most often applied in treatment for illicit substance use, harm reduction is increasingly used in many different settings, with a variety of populations, and in instances where there is a desire to reduce the negative effects of legal/licit substances, such as in tobacco smoking reduction and e-cigarette substitution programs [6, 7], in programs to reduce the harms associated with alcohol [6, 8, 9], in interventions addressing eating disorders or domestic violence [10], or with people who exchange sex for drugs, money, or material goods [11–13]. Nevertheless, harm reduction has not been formally incorporated into the daily repertoires of healthcare providers who aim to improve health behaviors (e.g., physical activity, nutrition) among their patients.

Harm reduction is sometimes met with resistance especially as it applies to people who use substances. Critics advocate for abstinence-only programs, arguing that anything less enables and encourages continued drug use and produces greater harm to the community. However, the benefits of harm reduction programs are clear. A systematic review of research on syringe exchange programs shows that they are cost-effective in reducing HIV transmission [14] and increase exchange users’ access to other medical and social support services [15]. Safe injection facilities have increased enrollment in detoxification treatment and have not been shown to increase social disorder in the community [16]. Housing first programs, in which services are provided to individuals without requiring that they abstain from substance use, reduce not only costs of medical and social care but also alcohol use [17] and have been shown to improve clinical outcomes for individuals living with HIV/AIDS [18]. The extant research provides a strong evidence base regarding the feasibility, effectiveness, and cost-effectiveness of harm reduction approaches [3, 6, 8, 9, 19–25].

The US-based Harm Reduction Coalition correctly notes that there is no single definition or formula for implementing harm reduction since harm reduction-informed approaches focus on specific individual and community needs [26]. However, Harm Reduction International (HRI) broadly describes harm reduction as “…policies, programmes and practices that aim primarily to reduce the adverse health, social and economic consequences of the use of legal and illegal psychoactive drugs without necessarily reducing drug consumption. Harm reduction benefits people who use drugs, their families and the community.” [27]. HRI has released a position statement describing the main characteristics of harm reduction for use with people who use all psychoactive drugs. These characteristics include (1) targeting risk and harms to people who use substances, understanding the roots of these risks, and tailoring interventions to reduce them; (2) acknowledging the significance of any positive change that people who use substances make in their lives; (3) accepting people who use drugs as they are and treating them with dignity and compassion; (4) protecting the human rights of people who use drugs; (5) and maintaining transparency in decisions about interventions as well as their successes and failures. In addition, HRI asserts the evidence base and cost-effectiveness of harm reduction approaches and underscores the importance of challenging policies and practices that are harmful to people who use substances (such as criminalization of substance use, lack of access to naloxone, and the denial of healthcare services to those who use substances) [27].

While harm reduction policies, programs, and practices in the context of substance use are well defined, they lack a broader applicability to non-drug-related harm. Given that a harm reduction approach can address other risk behaviors that often occur alongside drug use [5] and that harm reduction principles have been applied to harms as described above, a natural evolution of the harm reduction philosophy is to extend it to other health risk behaviors and to a broader healthcare audience. Thus, the aim of this paper is to build on existing literature and practice to specify and define a broad and applicable set of harm reduction principles that are generalized beyond substance use for operationalization in healthcare settings.

## Methods

This work is derived from a 2016 mixed methods study of an HIV clinic that provides harm reduction-informed services. This clinic demonstrates high rates of clinical success, with 74% of patients meeting standards set by the US Health Resources and Services Administration for retention in care [28], 95% of patients being prescribed antiretroviral therapy (ART), and 87% of patients virally suppressed. Included in these rates are patients in populations that typically experience poor clinical outcomes, such as those with housing instability, mental health diagnoses, and substance use disorders. Overall findings from our mixed methods evaluation study are reported elsewhere, including a report of patients’ clinical outcomes and an analysis of how the clinic environment contributes to those outcomes [29, 30]. Through discussions with clinic leadership, it became evident that they believed their harm reduction emphasis was a contributing factor to their patients’ clinical success. Our current study aimed to understand how HR was operationalized in this setting and to use these data to characterize operant principles of HR in general healthcare settings.

To address this aim, we examined the extant research and gray literature to conceptualize harm reduction as a philosophy of care, with emphasis on the characteristics of harm reduction described by HRI. Building on this foundation, we then developed qualitative interview protocols for patients and staff members to explore the ways harm reduction was applied in the clinic. We conducted one-on-one interviews with 23 patients and 17 clinic staff, including physicians, nurses, a nurse practitioner, a physician assistant, social workers, medical assistants, and administrative team members. We distributed flyers in the clinic to recruit patients, and staff members informed their patients of the opportunity to participate and the confidential and voluntary nature of participation. Patients who participated in interviews received $20 gift cards to a local grocery store to honor their time. Staff members were informed of the study, invited to participate, and assured of confidentiality during a monthly staff meeting. The study was approved by the Institutional Review Board of the hospital where the study took place and through the Human Research Protection Office of the university where the researchers worked.

Each interview was audio recorded and transcribed verbatim. We then analyzed and coded transcripts in NVivo (QSR International Pty Ltd. Version 10) using a deductive content analysis to understand the extent to which harm reduction concepts were supported by the data [31] as well as an inductive analytic approach [32, 33] to explore emerging themes. Specifically, four members of our study team reviewed the transcripts to contextualize key themes and then developed an initial code list. Next, three members of the team coded one of the transcripts line-by-line to test for consistent code application and to explore new themes. Discrepancies in coding were discussed by the team until consensus was reached, and new codes were also discussed and added to the codebook. Two researchers then used the revised codebook to review the remainder of the transcripts, and finally, the team used axial coding to review code co-occurrences and to understand how the themes related to each other [34].

We used the results of these data from our qualitative interviews to refine existing harm reduction concepts and develop harm reduction principles that may generalize to other healthcare settings, aiming for a set of principles that were comprehensive, succinct, and distinct from one another. We also sought to describe each principle using action words or phrases that were easily understood and thus more likely to be put into practice. In consultation with harm reduction experts, which included individuals leading national harm reduction efforts as well as those with decades of experience providing harm reduction-informed health care, we then refined each of the principles by discussing disagreements until reconciliation was reached. Here, we describe the resultant harm reduction principles for healthcare settings and provide a definition for each principle.

## Results

A summary of harm reduction principles, definitions, and approaches for healthcare providers is delineated in Table 1. The first principle that we derived from our data is *humanism*, a central underpinning of the harm reduction approach that describes the way that providers value, care for, respect, and dignify patients as individuals. This principle is demonstrated by the following quote from a patient:

*Well, they sit down and they talk to you and they tell you “Listen. You don’t have to be afraid. You don’t have to be afraid to tell us anything.” They open their hearts and they listen. …a lot of people treat HIV and AIDS patients the way you wouldn’ expect, you know, they treat them that they got their disease from being deceiving and evil and abominable. Every time people treat HIV and AIDS patients they look at them as drug addicts or homosexuals. They feed them to the lions, as far as conversation and trust. This place, they don’t treat you like an outcast, they listen, help. …I love them. This is like my second home. ….If a disaster happened in America and I wanted to check on family and friends, I would run up here and make sure these people were okay because I love them, they are good people*. [Patient 1].

**Table 1:** Harm reduction principles, definitions, and approaches for healthcare settings

Principle	Definition	Approaches
1. Humanism	• Providers value, care for, respect, and dignify patients as individuals. • It is important to recognize that people do things for a reason; harmful health behaviors provide some benefit to the individual and those benefits must be assessed and acknowledged to understand the balance between harms and benefits. • Understanding why patients make decisions is empowering for providers.	• Moral judgments made against patients do not produce positive health outcomes. • Grudges are not held against patients. • Services are user-friendly and responsive to patients’ needs. • Providers accept patients’ choices.
2. Pragmatism	• None of us will ever achieve perfect health behaviors. • Health behaviors and the ability to change them are influenced by social and community norms; behaviors do not occur within a vacuum.	• Abstinence is neither prioritized nor assumed to be the goal of the patient. • A range of supportive approaches is provided. • Care messages should be about actual harms to patients as opposed to moral or societal standards. • It is valuable for providers to understand that harm reduction can present experiences of moral ambiguity, since they are essentially supporting individuals in health behaviors that are likely to result in negative health outcomes.
3. Individualism	• Every person presents with his/her own needs and strengths. • People present with spectrums of harm and receptivity and therefore require a spectrum of intervention options.	• Strengths and needs are assessed for each patient, and no assumptions are made based on harmful health behaviors. • There is not a universal application of protocol or messaging for patients. Instead, providers tailor messages and interventions for each patient and maximize treatment options for each patient served.
4. Autonomy	• Though providers offer suggestions and education regarding patients’ medications and treatment options, individuals ultimately make their own choices about medications, treatment, and health behaviors to the best of their abilities, beliefs, and priorities.	• Provider-patient partnerships are important, and these are exemplified by patient-driven care, shared decision-making, and reciprocal learning. • Care negotiations are based on the current state of the patient.
5. Incrementalism	• Any positive change is a step toward improved health, and positive change can take years. • It is important to understand and plan for backward movements.	• Providers can help patients celebrate any positive movement. • It is important to recognize that at times, all people experience plateaus or negative trajectories. • Providing positive reinforcement is valuable.
6. Accountability without termination	• Patients are responsible for their choices and health behaviors. • Patients are not “fired” for not achieving goals. • Individuals have the right to make harmful health decisions, and providers can still help them to understand that the consequences are their own.	• While helping patients to understand the impact of their choices and behaviors is valuable, backwards movement is not penalized.

This concept also emerged in interviews with providers:

…*On one level I think that there is a sense of being respected and cared for and valued by the clinicians who patients see here. So that’s something that I hear people report to me…you know, like, “[name redacted] really listened,” or when, “he came to visit me after my diagnosis,” or you know, “[name redacted] helped me with this,” so you know, I get a sense that people feel very valued and they’ve said this explicitly to me*. [Provider 1].

In working with patients demonstrating a range of both harmful and healthful behaviors, providers recognized that patients behave the way they do for specific reasons. Behaviors that contribute to negative health outcomes were seen as providing some benefit to patients or these behaviors would not have occurred and sustained. Humanism includes providing services without moral judgements against patients, since these do not produce positive health outcomes.

The second principle, *pragmatism*, reflects the idea that none of us will ever achieve perfect health behaviors and that “perfect” health behaviors are impossible to define. While providers may have wished their patients could make healthy choices every time and in every situation, these providers recognized this was an unrealistic expectation. A pragmatic approach meant that abstinence from harmful health behaviors was neither prioritized nor assumed to be the goal of the patient; rather, a range of supportive approaches were offered.

*Everybody don’t get it on the first try, so I might miss my meds, but it’s like, they don’t throw it away. They say, “Oh, we got to start a new plan. Well let’s pick up from where you were. Let’s try this.” I think that’s been very helpful*. [Patient 2].

*We had a patient in [a neighborhood (name redacted)] and she, I think she did IV drug use every day of her life. And [the provider (name redacted)] was like, “Ok, I’m not gonna get you to stop, but if you can shoot up every day then you can take your meds before you shoot up.” Her mother called back and was like astonished and [the provider] was like, “Well, what did you want me to say to her? Don’t take your meds and die? Because that’s going to kill you before your heroin, or your whatever it is that you’re injecting. I have the same philosophy really. I don’t want you to do that, it’s illegal, it’s gonna get you in trouble, it could make you very sick, but you’re going to do it regardless of what I have to say; it’s your life. But what I can say is if you’re going to do it all the time then you can remember to take your meds, we do expect that. And it works, she’s undetectable to this day.”* [Provider 2].

Providers at this clinic also recognized that patients’ health behaviors and their abilities to change them were influenced by social determinants and community norms and by long histories of harmful health behaviors. These providers were able to respond pragmatically by focusing resources on health behaviors, such as ART adherence, that were most amenable to change and had high individual and public health impacts.

*Individualism* reflects the idea that every person presents with their own needs and strengths as well as with a spectrum of health behaviors and receptivity for intervention. In response to this philosophy, patients in the clinic were presented with a menu of intervention options. To identify the most helpful approaches for the individual, each patient’s needs and strengths were assessed and no assumptions were made based on their histories of harmful health behaviors.

*Well, I think really sitting down with the patient and finding out what’s going to work for them. Not everyone brushes their teeth every morning and evening. Not everyone drinks coffee every morning. Not everyone has a certain thing that they do every day and it’s just trying to figure out what that thing might be or if there isn’t a thing, what other way we can help, can we set an alarm? Can we give you a phone call? …Is there some person that you see every day that can be a coach for you? Or do you need to come in and see us every day, in order to remember to take your meds? So we do have a fair number of patients who are on, like a modified daily observed therapy, modified in that they come Monday through Friday and we kind of give them the weekend on the honor system, for a month. But really, like, just sitting down and trying to figure out what’s going to work best for the patient, sometimes pill box is the answer, and sometimes it’s not. Sometimes it’s something else*. [Provider 3].

In its messages to patients, the clinic did not convey that patients should be enabled to continue harmful health behaviors without an understanding of potential consequences, but rather educated patients about the actual risks that are associated with their behaviors.

The next harm reduction principle that emerged from our interviews is *autonomy*. Though one of the roles of health care and public health professionals is to improve patients’ health literacy by providing suggestions and education regarding patients’ treatment options, patients in the clinic ultimately made their own choices about medications, treatment, and health behaviors to the best of their abilities, beliefs, resources, and priorities.

*They will ask me, do I want to come to a meeting, do I want to do this, do I want to do that, what is a good time for me to come? It is, they always ask me, do I want to do or they ask me or give me suggestions.* [Patient 1].

*I think that if you approach a patient and say to them, “These are the reasons why you should do what I’m saying,” that patient maybe’s like, “Ok, doc, I’ll do that,” but if they come up with the reasons why instead of you telling them the reasons why, people are a lot more apt to change their behavior. If they’re able to come up with those ideas, look at the pros and cons…so if I didn’t take my HIV medicine, what bad can come out of it? Of course everybody knows that. I’m going to get sick, and I’m going to get all of these opportunist infections. Or what good would come out of it? Instead of me [listing potential positive outcomes], have them come up with the goods and bads. Hopefully what they do is they come up with multiple goods and bads and they say wait a second, yeah maybe I’ll do that. It’s kind of a different approach.* [Provider 4].

*Incrementalism* is a major tenet of harm reduction and refers to the idea that any positive change demonstrated by the patient is a step toward improved health and that positive health changes often can take months or years to achieve. By allowing patients to succeed in small ways and reinforcing progress toward their own goals, they remain engaged in care and have access to trusted providers in times of crisis or acute illness. Providers at the HIV clinic applied this principle by helping patients to celebrate any positive movement and viewed positive reinforcement as beneficial to the patient and the provider-patient relationship. Patients often expressed a sense of amazement when positive behaviors were celebrated, sometimes in very simple and concrete ways as described in the following patient quote:

*Patient*:…*not too long ago they gave me a card that let me know that I’m doing good! You know how you get birthday cards? They gave me one of them cards telling me I’m doing good, to stay up on [my HIV medications]. They make me feel a little good about myself. The whole staff does. Everybody wrote on the card. They gave me a card to let me know that I’m doing good.* [Patient 4].

The concept of “celebrating” improvements rather than “punishing” regression is also described by this provider:

*I think we build up people’s self-esteem and their self-efficacy…When somebody comes in, for instance, [and] says they’re so upset, they’re so despondent, they used crack last night and they fell off the wagon, and now the past six months are to shit and… dadadadada, [we reframe and ask], “What are the negative consequences you had? Oh you didn’t spend all your whole paycheck? You stopped sometime last night? You came in here this morning?” Like there’s a million things you did right. Whereas the majority of healthcare providers would be like, “See the failure and go with the failure, and what do you want to do to fix it?” So it really does take turning things on its head on a pretty regular basis, and it’s hard because the healthcare system just doesn’t think that way; that’s not the norm. If we waited until everybody stopped using before they accomplish whatever they want to accomplish a lot of people would be dead.* [Provider 5].

Finally, providers applied harm reduction principles by practicing *accountability without termination* as a critical component of care. In short, patients were seen as being responsible for their own health choices and outcomes but were never “fired” from care.

*Interviewer*: *I’m curious to know, how does the staff respond when you go off, as you said? How do they react to that?*

*Participant*: *Well…no one ever actually calls security on me. They’ve come close. I thought it was more important how they reacted afterwards.*

*Interviewer*: *Hmm, tell me about that.*

*Participant*: *Maybe the next day, everybody is trying to get me here again. That I appreciate because most times I’ll be full of guilt. I have a problem with apologizing. Every time I’ve done it, I’ve apologized. When you verbally assault someone like that, I kind of look at them the next day, you look at them hard and see how they’re treating you now. Are they treating you the same way and stuff like that? Everyone is given at least second and third chances. I mean genuine second and third chances. They actually don’t hold a grudge, they don’t appear to, you know what I mean? I’m sure there’s a question in their mind, is he gonna go off again? But they treat you with the same respect and concern. If it weren’t for that I wouldn’t be here*.

[Patient 5].

*People fuck up all the time and patients get scripts and divert them all the time, we know that that’s happening [but] we have to respect the law as well, so we don’t lie to people about it. I think that’s a big difference. We don’t [discount] things like pain and symptoms from people who have drug addictions and we don’t [discount] the importance of following laws that have to be followed. What I think we do differently is we don’t automatically assume anybody is doing anything wrong but we explain, “I have to do this and this is why, and I’m going to give you a heads up about that.” It’s not some way of exerting control and power, it’s part of a communication process with the patient and they can choose to walk away from that any time they want…A lot of other places, I think, patients just get fired. And we almost never fire people, ever*. [Provider 5]

## Discussion

Building on the critical work by HRI and others in the field, we used data from qualitative interviews with staff and patients of an HIV clinic to define a set of harm reduction principles for use in healthcare settings. Because most people engage in health behaviors unrelated to substance use that are harmful or suboptimal, harm reduction is appropriate for all patients and not just those who use illicit substances. Adopting harm reduction principles in healthcare settings can therefore benefit a wide range of patients. Thus, the primary means by which our work extends the characteristics of harm reduction as described by HRI is that we have described the principles in a way that broadens their application beyond work with people who use substances and operationalizes the use of these principles in healthcare settings.

The principle we defined as humanism is parallel to HRI’s reference to dignity and compassion. The decision-making balance between healthful and harmful behavioral balance is often tipped by short-term versus long-term benefits and priorities. Understanding these phenomena is important not only because it will likely improve the provider-patient relationship, but also because doing so is empowering to providers who might otherwise feel frustrated by working with people that chronically demonstrate harmful behaviors. In addition, it is empowering to patients who may feel validated that a provider has taken the time to understand the reasons behind their actions.

Pragmatism is consistent with HRI’s focus on targeting interventions to specific risks and harms. Messages about patient care must be specific to actual harms to patients as opposed to moral or societal standards. In addition, behaviors do not occur within a vacuum and therefore can be difficult to change at the individual level. In some cases, the “ideal” healthy behavior suggested by a provider may be initially achievable by the patient yet result in harm if the behavior is not sustainable in the social context of the patient. Pragmatism is a practical approach to problems that are short-term, concrete, and rooted in the experience and social context of the patient and tested in that context. This is important because providers may provide solutions that are not viable in the patient’s world or worldview and therefore not achievable or helpful, setting up a continuing cycle of blame and disempowerment for the patient. Short-term, realistic goals based in the patient’s context can provide quick benefit and establish agency in the patient, in turn reinforcing a positive working relationship with the provider. In a review of the literature, Rhodes et al. described social and structural factors that impact HIV risk for people who use intravenous drugs, which include but are not limited to neighborhood disadvantage, social capital, policy and policing, and the role of social stigma and discrimination [35]. Though we again note that this rich description is focused on people who use substances, these factors certainly have health implications across many fields of medicine. Therefore, a pragmatic approach not only targets actual harms but also considers the structural production of risk. While we confirm that the patient should be held accountable for his harmful health behaviors, understanding the underlying factors contributing to risk is critical to working successfully with patients.

We also included individualism as one of our principles. Though this is not specified by HRI as a characteristic of harm reduction, it is certainly consistent with HRI’s commitment to tailoring interventions to address risks of individuals. Individualism captures the idea that there is no universal application of protocol or messaging for patients but that providers must tailor interventions and messages to each person and maximize their options for supportive services (e.g., housing, employment assistance, substance use treatment).

Autonomy falls under HRI’s umbrella of universality and interdependence of rights. Patient-provider partnerships affect health outcomes for the patient, so these relationships are exemplified by patient-centered care, shared decision-making, and reciprocal learning. Health and healthcare decision-making is a set of care negotiations based on the current state and goals of the patient. Autonomy is akin to the health literacy model of care in which people have “…the capacity to obtain, process, and understand basic health information and services necessary to make appropriate health decisions” [36].

Incrementalism, which is also referred to in HRI’s description of harm reduction, underscores the importance of recognizing that all people at times experience plateaus or nadirs and that acknowledging this fact can help providers deal with the frustration they are likely to experience in these situations. It is important for providers to understand this and to plan for static or negative health trajectories.

Accountability without termination extends HRI’s reference to transparency, accountability, and participation. Previously, we noted that when patients participate in harmful health behaviors, providers must seek to understand the benefits to the individual that accompanies the harm. However, we also note that patients are responsible for their health choices and behaviors. Thus, when practicing harm reduction, providers do not “fire” patients for not achieving their individual health goals. While individuals have the right to make a range of harmful and healthful decisions about their care, providers can still help them understand that the consequences are their own. While helping patients to understand the impact of their choices and behaviors is valuable, backwards movement is not penalized. While it did not come through in our data, we also acknowledge that in its discussion of accountability, HRI correctly emphasizes that providers also must be accountable for their own decisions, success, and failures [27]. Additional research is needed to better understand how provider accountability affects patient outcomes in the context of harm reduction approaches to care.

Our findings are limited by the fact that they derive from qualitative interviews in one HIV clinic and have not yet been tested in other healthcare settings. It is not surprising that this work took place in an HIV clinic given that providers in this field tend to be more practiced in harm reduction as compared to other medical specialties. However, this fact also increases our confidence in the utility of the principles, given that they emerged from a harm reduction-informed medical setting. The single-site limitation is also mitigated by the facts that we interviewed patients and many different kinds of providers and that, in defining the principles, we referred to the work of others in the field, particularly Harm Reduction International and the UK Harm Reduction Alliance [37].

## Conclusions

We are among the first to provide a comprehensive set of principles for universal harm reduction as a conceptual approach for healthcare. While the principles are not discrete and overlap in some instances, we sought to describe them in a way that incorporates the foundation provided by HRI and that also enables the healthcare professional to consider and implement them separately. Although harm reduction has typically been conceptualized as a strategy for people who use substances, the reality is that patients with conditions such as obesity are similarly chronic and can benefit from a harm reduction approach. Because we believe that it is important to recognize harm reduction as a set of principles useful for work with a broad range of patients beyond those who use substances, we also include a set of examples in Table 2 that describe how each principle might be applied with patients with obesity. Applying harm reduction principles in health and healthcare settings may improve clinical care outcomes given that the quality of the provider-patient relationship is known to impact health outcomes and treatment adherence. Our proposed set of principles emphasizes shared decision-making between the provider and patient, which has been shown to improve patient satisfaction, clinical outcomes, and costs of care.

**Table 2:** Examples of application of harm reduction principles for patients who are obese

Principle	Example
1. Humanism	• Providers do not shame or think less of patients with obesity. • Providers strive to understand underlying factors contributing to patients’ obesity, which may include lack of access to healthy food or unhealthy eating habits that are rooted in family traditions or local culture. • Clinicians do not impose their personal beliefs about diet upon patients who are overweight or assume that weight loss is the patients’ prioritized goal.
2. Pragmatism	• Providers do not expect that the obese patient will never eat processed or sugary foods again. • Behavioral interventionists encourage patients to reduce their consumption of processed or high-fat, low-nutrition foods. • Rather than mandating that patients must lose a specific amount of weight, providers work with patients to establish realistic eating goals, which may or may not include weight goals.
3. Individualism	• In working with patients who are obese, providers might strive to understand the patient’s experience and how it contributes to suboptimal health, then offer appropriate interventions. For example, food vouchers or referrals to food pantries with fresh produce might be a useful support for patients without access to healthy food.
4. Autonomy	• In working with overweight patients, providers might assess readiness to lose weight and provide patients with health improvement education and options. • Providers support their patients in developing plans to implement health-promoting strategies that are acceptable to the patient, such as adding exercise intervals or incorporating fresh produce into their diets.
5. Incrementalism	• For the obese patient, any weight loss, increase in physical activity, or improvement in other clinical markers is seen as success. • For patients who overeat, healthy eating is viewed as an ongoing, gradual process. • For patients who are interested in losing weight, weight gain is not seen as failure but as part of the process.
6. Accountability without termination	• Patients who are overweight and have diabetes continue to receive insulin even though they regularly eat foods with high sugar content. • Patients who are obese are not terminated from care if they continue to gain weight.

Supporting patients who make harmful health choices may present moral ambiguity on the part of providers, particularly those who observe short-term negative outcomes. However, supporting patients’ choices and healthcare goals may not only provide emotional relief to providers who acknowledge that they are not solely responsible for “fixing” patients’ behaviors, but also provide long-term, albeit not always linear, improvement in patient outcomes and health costs by retaining patients in care.

This paper aims to further the discussion about the value of harm reduction, broadening the scope of this philosophy to inform care, develop policy, and apply it to a range of patient populations. Harm reduction can be thought of as a universal precaution and applied to all individuals regardless of their disclosure of negative health behaviors, given that health behaviors operate along a continuum and are not binary. It will be important to further operationalize methods of harm reduction-informed care, develop measures to assess each of the principles, and create and test mechanisms for implementation of this treatment philosophy. Finally, it will be critical to empirically test whether the harm reduction philosophy of care increases patient engagement and retention and improves clinical outcomes and healthcare costs.
